# Coevolutionary dynamics between tribe *Cercopithecini* tetherins and their lentiviruses

**DOI:** 10.1038/srep16021

**Published:** 2015-11-04

**Authors:** Junko S. Takeuchi, Fengrong Ren, Rokusuke Yoshikawa, Eri Yamada, Yusuke Nakano, Tomoko Kobayashi, Kenta Matsuda, Taisuke Izumi, Naoko Misawa, Yuta Shintaku, Katherine S. Wetzel, Ronald G. Collman, Hiroshi Tanaka, Vanessa M. Hirsch, Yoshio Koyanagi, Kei Sato

**Affiliations:** 1 Laboratory of Viral Pathogenesis, Institute for Virus Research, Kyoto University, Kyoto 6068507, Japan; 2 Department of Bioinformatics, Medical Research Institute, Tokyo Medical and Dental University, Tokyo 1138510, Japan; 3 Department of Medical Virology, Faculty of Life Sciences, Kumamoto University, Kumamoto 8608556, Japan; 4 Laboratory of Molecular Microbiology, National Institute of Allergy and Infectious Diseases, National Institutes of Health, Bethesda, Maryland 20892, USA; 5 Department of Microbiology, Institute of Health Biosciences, The University of Tokushima, Tokushima 7708503, Japan; 6 Wildlife Research Center, Kyoto University, Kyoto 6068203, Japan; 7 Japan Monkey Centre, Aichi 4840081, Japan; 8 Department of Medicine, University of Pennsylvania Perelman School of Medicine, Philadelphia, Pennsylvania 19104, USA; 9 CREST, Japan Science and Technology Agency, Saitama 3220012, Japan.

## Abstract

Human immunodeficiency virus, a primate lentivirus (PLV), causes AIDS in humans, whereas most PLVs are less or not pathogenic in monkeys. These notions suggest that the co-evolutionary process of PLVs and their hosts associates with viral pathogenicity, and therefore, that elucidating the history of virus-host co-evolution is one of the most intriguing topics in the field of virology. To address this, recent studies have focused on the interplay between intrinsic anti-viral proteins, such as tetherin, and viral antagonists. Through an experimental-phylogenetic approach, here we investigate the co-evolutionary interplay between tribe *Cercopithecini* tetherin and viral antagonists, Nef and Vpu. We reveal that tribe *Cercopithecini* tetherins are positively selected, possibly triggered by ancient Nef-like factor(s). We reconstruct the ancestral sequence of tribe *Cercopithecini* tetherin and demonstrate that all Nef proteins are capable of antagonizing ancestral *Cercopithecini* tetherin. Further, we consider the significance of evolutionary arms race between tribe *Cercopithecini* and their PLVs.

Based on the sequence similarity, the following two issues have been widely accepted: (i) human immunodeficiency virus type 1 (HIV-1), the causative agent of acquired immunodeficiency syndrome, emerged from zoonotic transmission of a simian immunodeficiency virus (SIV) in chimpanzee (SIVcpz) to humans around 100 years ago^1^,^2^,^3^; and (ii) SIVcpz appears to have emerged from the recombination of two lineages of SIVs from Old World monkeys (OWMs): SIVgsn/mon/mus lineage from greater-spot nosed monkey (*Cercopithecus nictitans*; GSN), mona monkey (*Cercopithecus mona*; MON), and mustached monkey (*Cercopithecus cephus*; MUS) and SIVrcm from red-capped mangabey (*Cercocebus torquatus*)^4^. Understanding the evolutionary history of primate lentiviruses (PLVs) including HIVs and SIVs is one of the most important and interesting topics in the field of retrovirology. However, because of their multiple cross-species transmissions and complicated recombination, it is difficult to elucidate how genetic conflicts between the ancient SIVs and their respective host species resulted in evolution and diversification.

OWMs, the family *Cercopithecidae*, are composed of 12 genera and a subfamily^5^. All SIVs identified so far encode 8 common genes: *gag, pol, env, tat, rev, vpr, vif*, and *nef *6. Among more than 40 SIVs, which have been identified in OWMs residing in Africa^7^, only 4 kinds of SIVs, SIVgsn in GSN, SIVmon in MON, SIVmus in MUS, and SIVden in Dent’s mona monkey (*Cercopithecus denti*) encode an additional accessory gene, *vpu*^8^. Importantly, the SIVs encoding *vpu* have been identified only in the monkeys belonging to tribe *Cercopithecini* including the genus *Cercopithecus*, strongly suggesting that the *vpu* gene has emerged in the evolution and transmission of SIVs in this tribe^5^,^7^,^8^.

To elucidate the co-evolutionary relationship between SIVs and their hosts, recent investigations have experimentally addressed the evolutionary conflict between viral and host proteins^8^,^9^,^10^ that stems from the “Red Queen hypothesis”^11^ or “evolutionary arms race” concept. Such an approach can be the way to explain the co-evolutionary history of SIVs and their host species. For example, Vif, a common protein encoded by all PLVs, has a robust ability to counteract a cellular anti-PLV restriction factor, apolipoprotein B mRNA editing enzyme catalytic polypeptide-like 3G^12^. In addition, another anti-PLV restriction factor, SAM domain and HD domain 1 (SAMHD1), can be antagonized by the viral accessory proteins, Vpr or Vpx^13^,^14^. The *vpx* gene is encoded in certain SIV lineages and HIV type 2, and it has been assumed that the *vpx* gene evolved from gene duplication of its ancestral gene, *vpr*^15^. Moreover, based on an experimental-phylogenetic investigation, Lim *et al.* recently proposed that the evolutionary interaction between Vpr/Vpx and SAMHD1 has undergone the following four steps: (i) Vpr acquired anti-SAMHD1 activity; (ii) ancestral SIV(s) created *vpx* by the gene duplication of *vpr*; (iii) Vpr transferred its anti-SAMHD1 activity to Vpx^16^. Namely, anti-SAMHD1 ability has been transferred from an old gene (*vpr*) to a new gene (*vpx*) during the co-evolution of SIVs and their hosts.

One of the most complicated examples of the co-evolutionary relationship between OWMs and their SIVs is tetherin (also known as bone marrow stromal antigen 2, CD317 and HM1.24) and its viral antagonists^8^,^17^. Tetherin inhibits the release of nascent viral particles from virus-producing cells^18^,^19^. Similar to the case of SAMHD1 and Vpr/Vpx, SIVs encode two kinds of anti-tetherin antagonists: Nef and Vpu^8^. Nef is encoded in all SIVs and most SIVs antagonize tetherins of their hosts by Nef^8^. On the other hand, as described above, Vpu is encoded in certain SIVs in OWMs, and the Vpu proteins of these SIVs potently antagonize tetherins of their hosts^8^,^20^. However, when, why, and how the *vpu* gene was acquired in certain SIV lineages during their evolution is still unclear.

In this study, we particularly focus on the OWMs belonging to the tribe *Cercopithecini* and their SIVs, and perform investigations based on molecular phylogenetics and evolution, experimental virology, and structural biology. We reveal that the tetherins of the tribe *Cercopithecini* are under strong positive selection. In addition, we construct the ancestral sequences of tribe *Cercopithecini* tetherin and experimentally demonstrate that all Nef proteins of the SIVs isolated from the tribe *Cercopithecini* retain antagonistic ability to the ancestral tetherin of tribe *Cercopithecini*. Moreover, we estimate the time of *vpu* acquisition in certain SIV lineages, and further, discuss the reason why *vpu* has been created and/or acquired from various scientific fields of view.

## Results

### Evolution of primate tetherin and CD4

**Figure 1:**
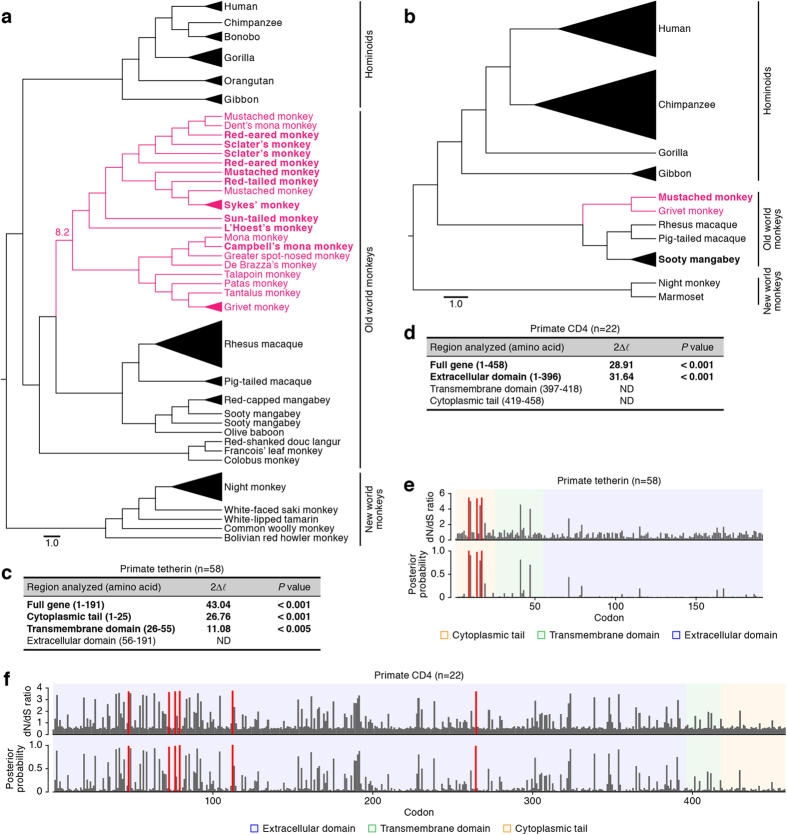
Molecular phylogenetic analyses of primate tetherin and CD4. (**a,b**) Phylogenic trees of 58 primate tetherins (**a**) and 22 primate CD4s (**b**) reconstructed using NJ method. Both trees were rerooted with the NWM clade. The species belonging to Tribe *Cercopithecini* are shown in pink. The species indicated in bold are the sequences newly identified in this study. GenBank accession numbers are listed in Tables 1 and 2. In panel a, the number (8.2) indicates the age of diversification (million years ago) that is estimated in a previous study^38^. A phylogenetic tree of 58 primate tetherins reconstructed using ML method is shown in Supplementary Fig. 1. (**c,d**) The positive selection detected in different regions of tetherin gene (**c**) and CD4 gene (**d**). The regions inferred to be under positive selection with statistical significance are represented in bold. ND, not detected. (**e,f**) Positively selected sites identified from tetherin gene (**e**) and CD4 gene (**f**). The codons under positive selection identified by PAML with posterior probability >0.95 are shown in red. All PAML analyses were performed under two models of codon usage, F61 and F3×4, and they yield consistent results.

Since lentiviral Nef and Vpu proteins have the common ability to down-regulate tetherin as well as CD4^17^,^19^,^20^,^21^, we set out to perform molecular phylogenetic analyses on primate tetherin and CD4. In this study, we newly identified 11 tetherin sequences of 8 different OWMs belonging to the tribe *Cercopithecini* (1 Campbell’s mona monkey, 1 mustached monkey, 2 Sclater’s monkeys, 1 L’Hoest’s monkey, 2 Sykes’ monkeys, 2 red-eared monkeys, 1 red-tailed monkey, and 1 sun-tailed monkey; listed in Table 1) and 3 CD4 sequences of 2 different OWMs (1 MUS and 2 sooty mangabeys; listed in Table 2). As shown in Fig. 1a,b, each family or infraorder (i.e., Hominoids, OWMs, or NWMs) respectively formed a monophyletic cluster on the reconstructed trees of both tetherin and CD4. On the other hand, within the cluster of *Cercopithecini*, the tetherins of certain *Cercopithecini* monkeys, particularly mustached monkey, red-eared monkey, and Sclater’s monkey, did not form a monophyletic subcluster, respectively (Fig. 1a). This indicates that the nucleotide sequence of certain *Cercopithecini* tetherins, particularly mustached monkey, Red-eared monkey, and Sclater’s monkey, are highly similar.

To detect positive selection in the evolution of primate tetherin and CD4, we estimated the nonsynonymous to synonymous (dN/dS) ratios. The two pairs of site models in PAML produced similar results and the results obtained from M7 (neutral model) versus M8 (selection model) comparisons are shown in Fig. 1c,d. Consistent with previous reports^22^,^23^,^24^ including ours^25^, the dN/dS ratio of primate tetherin was significantly greater than one for full-length (43.04), cytoplasmic tail (CT; 26.76), and transmembrane domain (TMD; 11.08) (Fig. 1c). Also, three codons, 9 (dN/dS = 5.2), 14 (dN/dS = 5.1), and 17 (dN/dS = 5.2) in primate tetherin, were identified to be positively selected sites with posterior probability greater than 0.95 (Fig. 1e). These findings on primate tetherin indicate that the functionally important regions of primate tetherin, particularly CT and TMD, have evolved under strong positive selection, which is in agreement with previous reports^22^,^23^,^24^,^25^.

On the other hand, the dN/dS ratio of primate CD4 was significantly greater than one for full-length (28.91) and the extracellular domain (ECD; 31.64), and six codons in the ECD, 48 (dN/dS = 3.6), 73 (dN/dS = 3.6), 77 (dN/dS = 3.6), 80 (dN/dS = 3.7), 113 (dN/dS = 3.7), and 265 (dN/dS = 3.7), were identified as positively selected codons by the site model analysis (Fig. 1f). These findings on primate CD4 suggest that the ECD of primate CD4 has evolved under strong positive selection. Because the ECD of CD4 molecule is homologous to immunoglobulins and plays a crucial role for immune recognition and immune responses^26^, the evolution and diversification of the immune system may closely associate with the positive selection observed in this study (Fig. 1d,f). In contrast to primate tetherin, positive selection was detected in neither TMD nor CT of primate CD4 (Fig. 1d,f). To down-regulate these cellular proteins, Nef targets the CTs of tetherin and CD4^21^,^27^, whereas Vpu targets the TMD of tetherin and the CT of CD4, respectively^8^,^28^. Therefore, our findings suggest that primate tetherin but not CD4 has experienced the positive selection elicited by Nef and/or Vpu during evolution.

### Positive selection detected in the evolution of tribe *Cercopithecini* tetherin

**Figure 2:**
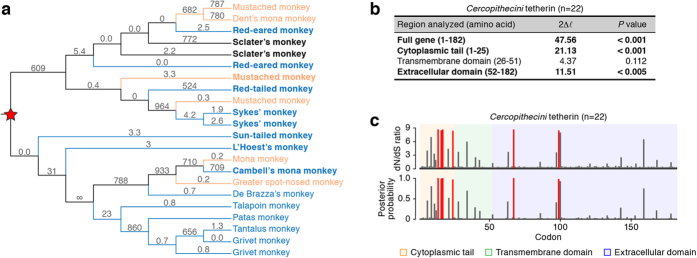
Molecular phylogenetic analyses of tribe *Cercopithecini* tetherin. (**a**) Phylogenic tree of 22 Tribe *Cercopithecini* tetherins reconstructed using NJ method. The species indicated in bold are the sequences newly identified in this study. The hosts of *vpu*-positive SIV are shown in orange, and those of *vpu*-negative SIV are shown in cyan. The numbers indicate the dN/dS value for each branch inferred by the branch model in the PAML analysis. The red star indicates the ancestral tetherin of tribe *Cercopithecini*. (**b,c**) The positive selection detected in different regions of tetherin gene. In panel b, the regions inferred to be under positive selection with statistical significance are represented in bold. In panel (**c**), positively selected sites identified by the PAML analysis are shown. The codons under positive selection identified by PAML with posterior probability >0.95 are shown in red. All PAML analyses were performed under two models of codon usage, F61 and F3x4, and they yield consistent results.

Among the SIVs in OWMs (identical to the family *Cercopithecidae*), *vpu*-positive SIVs have been identified only in the monkeys belonging to the tribe *Cercopithecini* (represented in pink in Fig. 1a,b). To elucidate the evolutionary interplay between Nef/Vpu and tetherin, we particularly focused on the tetherins of this tribe. The phylogenetic tree of 22 tetherins belonging to the tribe *Cercopithecini* showed that the tetherins of the hosts of *vpu*-positive SIVs intermingle with those of *vpu*-negative SIVs (Fig. 2a), suggesting that the presence of *vpu* did not result in the convergent evolution of *Cercopithecini* tetherin. Also, the site model analysis revealed that the dN/dS ratio of *Cercopithecini* tetherin was significantly greater than one for full-length (47.56) and CT (21.13) as well as ECD (12.15) (Fig. 2b). Four codons in the CT, 14 (dN/dS = 8.6), 16 (dN/dS = 8.5), 17 (dN/dS = 8.6), and 24 (dN/dS = 8.4), and two codons in the ECD, 67 (dN/dS = 8.3) and 99 (dN/dS = 8.4), were identified to be positively selected (Fig. 2c).

**Figure 3:**
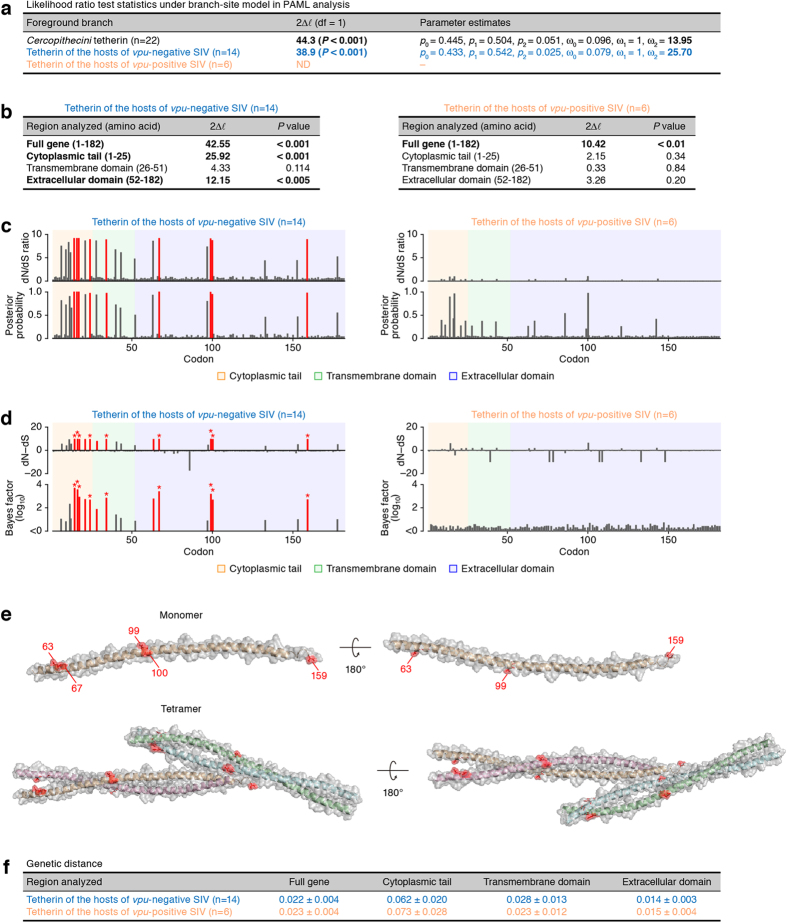
Molecular phylogenetic and structural analyses of tetherins of SIV-infected monkeys. (**a**) The result obtained from the three branch-site analyses for Tribe *Cercopithtecini* (n = 22), the hosts of *vpu*-negative SIV (cyan, n = 14), and those of *vpu*-positive SIV (orange, n = 6). The clades inferred to be under positive selection with statistical significance are represented in bold. (**b**) The positive selection detected in different regions of tetherin gene of the hosts of *vpu*-negative SIV (left, n = 14) and those of *vpu*-positive SIV (right, n = 6). The regions inferred to be under positive selection with statistical significance are represented in bold. (**c,d**) Positively selected sites identified in our analyses. In panel (**c**), the codons under positive selection identified by PAML with posterior probability >0.95 are shown in red. In panel d, the codons under positive selection inferred by HyPhy with Bayes factor >50 are shown in red, and the codons identified as positively selected sites by PAML are indicated with asterisks. (**e**) Structure modeling of the ancestral tetherin of tribe *Cercopithtecini*. The transparent surface with the ribbon diagram of the extracellular domain (ECD) of ancestral *Cercopithtecini* tetherin, which is generated by SWISS-MODEL server based on the ECD of human tetherin (PDB code: 3MQB)^31^, is shown. The two views of monomer (top) and tetramer (bottom) models, rotated by 180**°**, are respectively shown. The 5 positively selected sites in the ECD of tetherins of *vpu*-negative SIV hosts (codons 63, 67, 99, 100, and 159) are indicated in red. (**f**) Genetic diversity analysis. The values indicate the overall mean genetic distance, which is calculated by using Tamura-Nei model^56^ in MEGA6, with standard error.

We then classified 22 *Cercopithecini* tetherins into two groups: the hosts of *vpu*-positive SIVs and those of *vpu*-negative SIVs. Because SIV has not been identified in Sclater’s monkeys^29^, we excluded the tetherin sequences of 2 Sclater’s monkeys from this classification. As shown in Fig. 3a, the branch-site tests in PAML revealed that the likelihood ratio test was significant with *P* < 0.01 in the analysis of the tetherins of the hosts of *vpu*-negative SIVs as well as 22 *Cercopithecini* tetherins, suggesting that positive selection has most likely operated on the tetherins of the monkeys infected with *vpu*-negative SIVs. Also, the site model revealed that the dN/dS ratio of the tetherins of the hosts of *vpu*-negative SIVs was significantly greater than one for full-length (42.55), CT (25.92), and ECD (12.15) (Fig. 3b, left), and nine codons positioned at 14, 16, 17, 24, 34, 67, 99, 100, and 159 were identified to be positively selected (Fig. 3c. left). In addition, thirteen codons including the nine codons detected by the site model (indicated by asterisks in Fig. 3d) were identified as positively selected sites by the random effects likelihood (REL) analysis implemented in the HyPhy package with Bayes factor greater than 50 (Fig. 3d, left). Furthermore, we constructed the ancestral sequence of the 22 tetherins of tribe *Cercopithecini*, using the codeml program in PAML (indicated by a red star in Fig. 2a. The sequence information is available in Supplemental dataset) and constructed the structure homology model of the ECD of ancestral *Cercopithecini* tetherin (Fig. 3e). By mapping the five positively selected sites in the ECD (positioned at 63, 67, 99, 100, and 159), we found that these amino acids were located on the same aspect of the alpha helix structure (Fig. 3e).

In contrast to the tetherins from the hosts of *vpu*-negative SIVs, it was notable that positive selection was not detected in the tetherins from the monkeys infected with *vpu*-positive SIVs by the branch-site model (Fig. 3a) and REL analyses (Fig. 3d, right). Although the site model showed that the dN/dS ratio of the tetherins of the hosts of *vpu*-positive SIVs was significantly greater than one for full-length (2Δ*l* = 10.42, *P* < 0.01) (Fig. 3b, right), no positive selection was detected at the significant level (*P* < 0.05) for respective domains (Fig. 3b, right) and codons (Fig. 3c, right), which basically agreed with the result obtained from the branch-site test (Fig. 3a). To ask whether the difference in the positive selection between these two groups could be attributed to the difference in the number of tetherin sequences included, we performed genetic diversity analyses using MEGA6^30^. As shown in Fig. 3f, the genetic diversity of tetherins of these two groups was comparable. Taken together, these results indicate that the much weaker selective pressure detected for the tetherins of the hosts of *vpu*-positive SIVs is not likely due to the smaller sample size of this group, but suggesting that Vpu did not exert a strong selective pressure on the tetherins of the hosts of *vpu*-positive SIVs.

### Antagonism of *Cercopithecini* tetherin by SIV Nef

**Figure 4:**
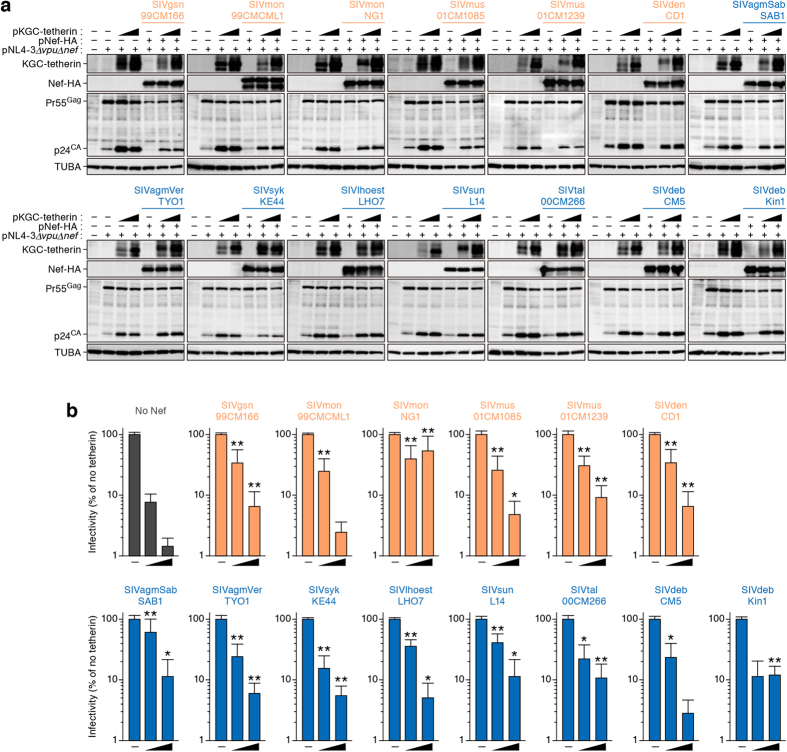
Experimental analyses of the anti-viral activity of ancestral *Cercopithtecini* tetherin and antagonistic ability of SIV Nefs. (**a**) Western blotting of cell lysates. Representative results are shown. Blots have been cropped; full uncropped blots are available as Supplementary Fig. 3. (**b**) TZM-bl assay. The data represents the percentage of infectivity compared to the values without tetherin with standard deviation. The assay was performed in triplicate. The statistic differences (*P < 0.05; **P < 0.01) versus the values of No Nef are determined by Student’s *t* test.

To directly evaluate the anti-viral activity of *Cercopithecini* tetherin and the antagonistic ability of SIV Nef proteins, we prepared an expression plasmid for the constructed ancestral *Cercopithecini* tetherin. Western blotting (Fig. 4a) and TZM-bl assay (Fig. 4b) revealed that increasing amounts of the ancestral *Cercopithecini* tetherin resulted in a dose-dependent decrease in the release of nascent virions. We confirmed that the infectious virus in the culture supernatant correlated strongly with the amount of supernatant viral p24 antigen, a physical measure of virion content (*r* = 0.947, *P* = 0.000031; Supplementary Fig. 2), which is consistent with previous reports^20^,^22^,^31^ and validates the tetherin impact on virus release.

We then prepared expression plasmids for 14 strains of SIV Nefs, which have been identified in the 12 species of *Cercopithecini* monkeys so far, and investigated whether these Nef proteins have the ability to antagonize the ancestral *Cercopithecini* tetherin. As shown in Fig. 4a, none of the Nef proteins affected expression levels of tetherin and Gag, particularly Gag precursor (Pr55^Gag^; Fig. 4a), which is consistent with previous reports^22^,^25^,^27^. Moreover, we revealed that all Nef proteins enhanced viral release in the presence of the ancestral *Cercopithecini* tetherin (Fig. 4b). Importantly, the 6 Nef proteins of *vpu*-positive SIVs significantly augmented viral release (Fig. 4b). These findings directly demonstrate that all SIV Nef proteins isolated from the tribe *Cercopithecini* potently antagonize *Cercopithecini* tetherin regardless of whether or not they had acquired *vpu*.

### Genetic and geographical consideration of the acquisition of *vpu* gene

**Figure 5:**
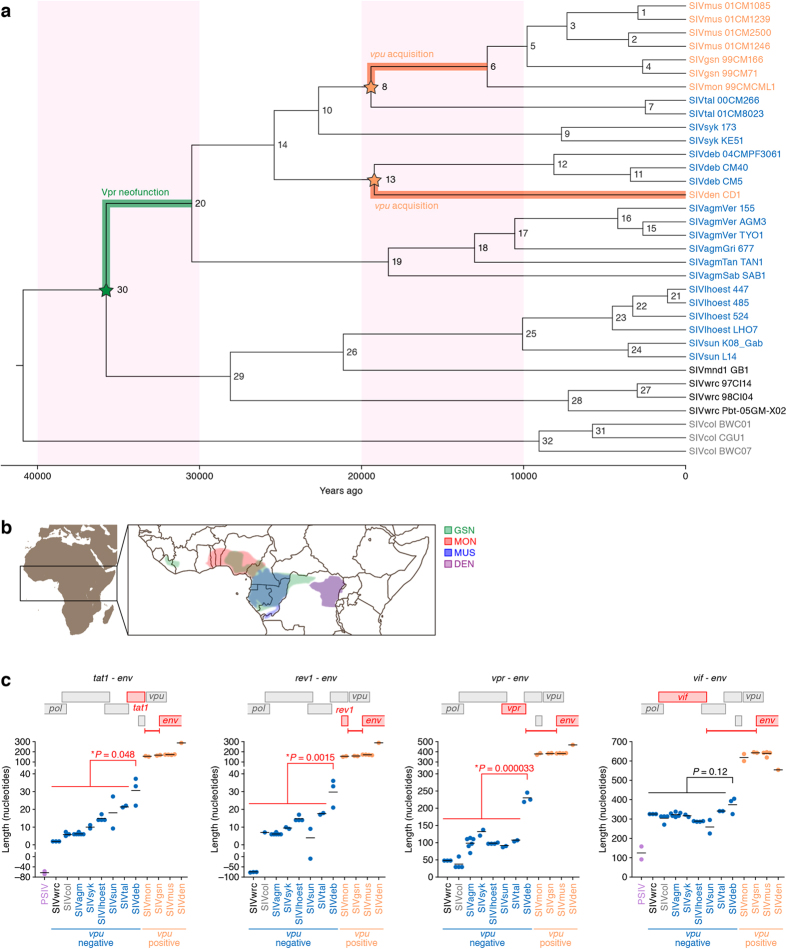
Evolution and diversification of SIV. (**a**) Dating the divergence times of 34 SIV lineages. The MCC tree constructed using BEAST is shown. This analysis was conducted by using the amino acid sequences of Gag, Pol, Vif, and Env. Cyan, *vpu*-negative SIVs; orange, *vpu*-positive SIVs; black, SIVs identified in western red colobus; and grey, SIVs identified in black-and-white colobus. The orange stars (nodes 8 and 13) indicate the time of *vpu* gene acquisition, and the green star (node 30) indicates the time of Vpr neofunction. X-axis indicates the year before present. GenBank accession numbers of the SIV sequences used in this analysis are listed in Table 4. The estimated divergence time, posterior probability, and bootstrap value of each node of the tree are listed in Table 3. (b) Distribution of the monkeys infected with *vpu*-positive SIV. The data is extracted from the reference^46^. The image is created using Illustrator (Adobe) by overlaying the maps shown in reference^46^. GSN, greater spot-nosed monkey; MON, mona monkey; MUS, mustached monkey; DEN, dent’s mona monkey. (**c**) The nucleotide length of SIV. The nucleotide length between the end of each viral gene (*tat1, vpr, rev1*, and *vif*) and the initiation codon of *env* are measured. Statistic differences between SIVdeb and the other *vpu*-negative SIV are determined by Welch’s *t* test. PSIV, prosimian endogenous lentivirus.

To further assess the possibility that Vpu has exerted selective pressure on the tetherins of tribe *Cercopithecini*, we performed a Bayesian evolutionary analysis for dating the time of *vpu* gene acquisition. As shown in Fig. 5a, our analysis revealed that the *vpu* gene has been independently acquired twice, which were estimated to have occurred in SIVgsn/mon/mus lineage (nodes 8) around 19,418 years ago and in SIVden lineage (node 13) around 19,218 years ago, respectively (Table 3). In addition, it has been reported that the gain-of-function of Vpr to degrade SAMHD1 occurred after the divergence from SIVsun/lhoest lineages^16^. Our analyses revealed that the Vpr neofunction occurred after 35,766 years ago (Fig. 5a and Table 3). These findings suggest that *vpu* was acquired by the two lineages of SIVs around 20,000 years ago, which were relatively recent events in the evolutionary history of SIVs compared to the neofunction of Vpr.

We then considered the acquisition of *vpu* gene in geographic terms. As considered in the previous papers^29^,^32^,^33^, the habitats of the three species of OWMs, GSN, MON, and MUS overlapped in West Africa including Cameroon, Gabon, and Nigeria, and Republic of the Congo, while the habitat of DEN was geographically separated (Fig. 5b). Therefore, it is plausible that *vpu* gene has been independently acquired by certain SIV(s) infecting the monkeys in these two separate geographic areas.

Furthermore, we considered the event of *vpu* acquisition in terms of viral genetics. Because of the restricted genome size of RNA viruses including lentiviruses, ‘genome compression’, which is caused by the use of overlapping genes, is a known characteristic of RNA viruses^34^. In fact, the 3′ end of all *vpu* genes of PLVs overlaps with the 5′ end of *env* (Fig. 5c, top). Although a previous study has suggested that there is no preference in the direction of frameshift in newly acquired viral genes^34^, we found that all *vpu* genes of OWM SIVs involved + 1 (forward) frameshift compared to *env* (data not shown). This suggests that the manner of *vpu* acquisition may be common in the two lineages of *vpu*-positive SIVs (Fig. 5a). Moreover, we measured the nucleotide length between the 3′ ends of 4 viral genes (*tat1, rev1, vpr*, and *vif*) and the 5′ end of *env*, where *vpu* is encoded in certain SIVs. As expected, the nucleotide lengths of these 4 regions in *vpu*-positive SIVs were clearly longer than those in *vpu*-negative SIVs (Fig. 5c). However, it was of interest that the nucleotide lengths from the 3′ ends of *tat1, rev1*, and *vpr*, but not of *vif*, in the 3 strains of SIVdeb were significantly longer than those of the other *vpu*-negative SIVs (Fig. 5c). Although the mechanism of new gene acquisition by viruses is still unknown^34^,^35^, these findings suggest that the nucleotide length in this region can vary in *vpu*-negative SIVs, and that this genomic region may be adequate for SIVs to create and/or acquire new gene(s).

## Discussion

In this study, we newly determined 11 tetherin sequences of the 8 species of the tribe *Cercopithecini* as well as 3 CD4 sequences of OWMs. In addition, we performed the in-depth molecular phylogenetic analyses and revealed that the tetherins of the tribe *Cercopithecini*, particularly those of the host monkeys of *vpu*-negative SIVs, are under strong positive selection. Furthermore, we constructed the ancestral sequence of tribe *Cercopithecini* tetherin and demonstrated that the ancestral *Cercopithecini* tetherin has a robust ability to inhibit viral release. In this regard, since a previous paper has shown that the “artificial” tetherin, which artificially forms the same topology to tetherin, sufficiently confers anti-viral activity^36^, it might not be so surprising that the ancestral *Cercopithecini* tetherin estimated in this study exhibited anti-viral ability. Nevertheless, here we demonstrated that the anti-viral activity of the ancestral *Cercopithecini* tetherin is strongly antagonized by all SIV Nef proteins we used. Particularly noteworthy was that Nef’s antagonistic activity against *Cercopithecini* tetherin is not associated with the presence of *vpu* gene. Moreover, we estimated the time of *vpu* gene acquisition in certain SIVs and further considered its significance.

Among the order Primates, the domains of tetherin and CD4 under positive selection differed: primate tetherin has been under positive selection in the CT and TMD, whereas the ECD of primate CD4 was positively selected (Fig. 1). These findings suggest that the factors triggering selective pressure on these molecules differ from each other. In the case of CD4, the ECD plays critical roles in immune control such as the recognition of major histocompatibility complex class II^26^. Moreover, CD4 is utilized for the invasion of PLV through interacting its ECD with their envelope glycoprotein (Env)^26^. Therefore, it is plausible that the ECD of CD4 has been positively selected through the immune pressures outside of the cells and the interaction with PLV Env^26^,^37^. Moreover, positive selection was detected in neither TMD nor CT of primate CD4 (Fig. 1d,f), suggesting that these domains are evolutionary stable. It is known that both Nef and Vpu target CD4 CT for the down-regulation^8^,^21^,^27^. Therefore, these findings suggest that primate CD4 has not been under positive selection caused by Nef, Vpu, and their ancestors. To the best of our knowledge, this is the first study evaluating the selective pressure on primate CD4 gene in-depth.

It was reported that the tribe *Cercopithecini* diversified approximately 8.2 million years ago (indicated in Fig. 1a)^38^. On the other hand, here we revealed that the *vpu* gene has been acquired in certain SIV lineages around 20,000 years ago (Fig. 5a and Table 3). These findings strongly suggest that the Vpu proteins encoded by the present-day SIVs cannot be the source of the selective pressure on *Cercopithecini* tetherin. In addition, the convergent evolution was not observed on the tetherins of *vpu*-positive SIV hosts (Fig. 2a). Furthermore, it was surprising that significant positive selection was not detected in any domains and codons of the tetherins of *vpu*-positive SIV hosts (Fig. 3a–d). Since there was no positive selection detected on the TMD of *Cercopithecini* tetherin (Fig. 2b), these findings suggest that ancestral Vpu-like factor(s) were not the selective pressure on the tetherins of tribe *Cercopithecini.*

In contrast to the tetherins of *vpu*-positive SIV hosts, the tetherins of the monkeys infected with *vpu*-negative SIVs exhibited strong positive selection in the CT and ECD (Fig. 3b–d). Interestingly, four out of the five positively selected sites in the ECD are located on the same aspect of its alpha-helix structure (Fig. 3e). This observation is reminiscent of the four amino acids, I34, L37, L41, and T45, in the TMD of human tetherin^28^. We have previously reported that these four amino acids in the TMD of human tetherin are responsible for HIV-1 Vpu-mediated antagonism and are located on the same helical face of the TMD^28^. Further study has revealed that the four amino acids in the TMD of human tetherin form the helix-helix intermolecular interaction with HIV-1 Vpu^39^. These findings raise the possibility that the four amino acids located on the same face of ECD may be positively selected from pressure caused by the other viral antagonists of tetherin. In fact, it has been revealed that the glycoprotein of Ebola virus, which sporadically causes epidemics in humans and primates residing in the central African countries such as the Democratic Republic of the Congo (formerly Zaire) and Sudan^40^, interacts with and antagonizes tetherin^41^. Moreover, Env of certain lentiviruses potently antagonize tetherin^42^,^43^,^44^,^45^. Therefore, these viral antagonists of tetherins and/or their ancestors could have exerted the selective pressure on the ECD of *Cercopithecini* tetherin. Moreover, although the main habitat of *vpu*-positive SIV hosts is West Africa (Fig. 5b), the monkeys infected with *vpu*-negative SIVs reside in a broad area of Africa including the central African countries^46^. This further suggests that the tetherins of *vpu*-negative SIV hosts have had the opportunity to be exposed to different pressures compared to those of *vpu*-positive SIVs.

As shown in Fig. 4b, we demonstrated that the infectivity of culture supernatant was significantly suppressed by the ancestral *Cercopithecini* tetherin. These findings suggest that the ancestral *Cercopithecini* tetherin possesses a robust activity to impair viral release and that the anti-viral ability of tetherin has been maintained in its evolution. On the other hand, it was surprising that all SIV Nef proteins including those of *vpu*-positive SIVs (e.g., SIVgsn, SIVmon, SIVmus, and SIVden) were capable of antagonizing *Cercopithecini* tetherin-mediated anti-viral ability (Fig. 4). It has been demonstrated that the Vpu proteins of *vpu*-positive SIVs antagonize tetherins of their natural hosts^20^. Therefore, these observations suggest that the *vpu* gene was not necessarily been acquired by certain SIVs to gain a novel anti-tetherin antagonist. In the case of the evolutionary interplay between Vpr/Vpx and SAMHD1, three evolutionary steps have been proposed: (i) acquisition of anti-SAMHD1 activity by ancestral Vpr (i.e., Vpr neofunction); (ii) creation of *vpx* by gene duplication in certain SIV lineages; and (iii) transfer of anti-SAMHD1 activity from Vpr to Vpx^16^. In contrast to the scenario of Vpr/Vpx and SAMHD1, our findings suggest that Nef proteins of *vpu*-positive SIVs have not lost their anti-tetherin activity even though a new tetherin antagonist, Vpu, was acquired, and that the transfer of anti-tetherin activity from Nef to Vpu has not occurred. Moreover, here we estimated that Vpr neofunction occurred around 36,000 years ago, which is relatively older than the acquisition of the *vpu* gene (Fig. 5a). Therefore, these findings imply that Nef still maintains anti-tetherin activity in *vpu*-positive SIVs because *vpu* is relatively younger than *vpr/vpx*, and that Nefs of *vpu*-positive SIVs may transfer their anti-tetherin activity to Vpu in the future.

As shown in Fig. 5a, our results suggest that the *vpu* gene was independently acquired in two SIV lineages: SIVgsn/mon/mus and SIVden. This raises three possibilities. First, it might be possible that the *vpu* gene acquired in SIVgsn/mon/mus lineage has been horizontally transferred to SIVden lineage and *vice versa*. Because the two virus lineages, SIVgsn/mon/mus and SIVden, share strikingly similar genomic features (e.g., very similar *vpu* genes with nearly identical locations), the most parsimonious explanation is that the acquisition of *vpu* gene was not independent events, but rather descended from a single original event. However, SIVgsn/mon/mus is phylogenetically divergent from SIVden (Fig. 5a), and the habitats of the monkeys infected with these two SIV lineages are geographically separated (Fig. 5b)^46^. These two notions argue against this first hypothesis. Second, there is a possibility that the *vpu* gene was acquired in the common ancestor of these two SIV lineages (i.e., node 14 of Fig. 5a) and then certain SIVs (e.g., SIVtal, SIVsyk, and SIVdeb) lost their own *vpu.* It might be possible for some viruses to lose the *vpu* gene because there are other routes to overcome OWM tetherin (e.g., Nef, Env)^8^. If something similar happened in an ancestral virus, it would relieve selective pressure to maintain a *vpu* gene. The third possibility is that the acquisition of *vpu* independently occurred twice in the two SIV lineages, perhaps the most feasible possibility. Further, it should be notified that the molecular clock analyses shown herein rely on the accessible information to date. It means that the information obtained in the future may affect the estimated age of *vpu* acquisition. Nevertheless, this is the first study inferring the time of accessory gene acquisition/generation by PLVs.

So, how was the *vpu* gene acquired and/or created ? Because the genome size of RNA viruses is strictly restricted, it has been assumed that RNA viruses evolutionary repeat trial-and-error to obtain new favorable genes^34^,^35^. Interestingly, we found that the nucleotide lengths between the 3′ end of *tat1* and the 5′ end of *env* of prosimian endogenous lentiviruses (PSIVs), which share a common ancestor with modern SIVs^47^,^48^, were much shorter than those of SIVs (Fig. 5c). In this regard, PSIVs encode *dUTPase* in *pol* region, while PLVs including SIVs do not^6^. This raises a possibility that the loss of *dUTPase* in SIV ancestors has relaxed the restriction of viral genome space, which allowed viruses to acquire and/or create novel genes. In fact, PLVs encode multiple viral genes around the region encoding *vpu* more than the other lentiviruses, which encode *dUTPase*^6^,^10^. Therefore, it is plausible that PLVs have gained the chance to acquire and/or create new genes by losing *dUTPase*, and that the acquisition of *vpu* might be one of the consequences.

## Methods

### Ethic statement

To determine the sequence of *tetherin*, blood was collected from wild-caught monkeys: 1 Campbell’s mona monkey (*Cercopithecus campbelli*), 1 mustached monkey (*Cercopithecus cephus*), 2 Sclater’s monkeys (*Cercopithecus sclateri*), 1 L’Hoest’s monkey (*Cercopithecus lhoesti*), 2 red-eared monkeys (*Cercopithecus erythrotis*), 1 red-tailed monkey (*Cercopithecus ascanis*), and 1 sun-tailed monkey (*Cercopithecus solatus*) according to the Guide for the Care and Use of Laboratory Animals^49^ under a NIAID Animal Care and Use Committee-approved protocol^50^,^51^. These procedures were approved by NIAID.

### Sequencing PCR

Genomic DNA was extracted from cryopreserved peripheral blood mononuclear cells (PBMCs) of these 9 monkeys^50^,^51^ by using DNeasy kit (Qiagen). Also, genomic DNA was extracted from the body hair root of 2 Sykes’ monkeys (*Cercopithecus albogularis*), which are kept in the Japan Monkey Centre, Inuyama, Aichi, Japan) by using DNA Extractor FM kit (Wako). PCR was performed by using PfuUltra High Fidelity DNA polymerase (Agilent Technologies) and the following primers: 5′-CAG CTA GAG GGG AGA TCT GGA TG-3′; 5′-CTC ACT GAC CAG CTT CCT GGG-3′, which were used in our previous study^25^. The obtained PCR products were purified by gel extraction and directly sequenced by using BigDye Terminator v3.1 cycle sequencing kit (Applied Biosystems) with the two primers described above and the following 4 primers: 5′-GGA CTT CAC CAG ACC CTG AA-3′; 5′-TTC AGG GTC TGG TGA AGT CC-3′; 5′-TCT CTC CTT TGC TCC CAA AA-3′; 5′-TTT TGG GAG CAA AGG AGA GA-3′. To determine the sequence of *CD4*, RNA was extracted from the cryopreserved PBMCs by using RNeasy Mini Kit (Qiagen). Reverse transcription was performed by using Thermoscript RT-PCR System (Life Technologies), and RT-PCR was performed by using Platinum *Taq* DNA polymerase High Fidelity (Life Technologies) and the following primers: 5′-CAG CAA GGC CAC AAT GAA C-3′ and 5′-TGC CTC AAA TGG GGC TAC-3′. The obtained RT-PCR product was purified by gel extraction and then cloned by using TOPO TA Cloning Kit (Life Technologies). The sequencing PCR was performed by using ABI Prism 3130 *xl* genetic analyzer (Applied Biosystems), and the data was analyzed by Sequencher v5.1 software (Gene Codes Corporation).

### Molecular phylogenetic analyses

The molecular phylogenetic analyses were performed as previously described^25^. Briefly, the 11 tetherin sequences newly identified in this study were aligned with 47 primate tetherin sequences (listed in Table 1) by using ClustalW implemented in MEGA6^30^. Also, the 3 CD4 sequences newly identified in this study were aligned with 19 primate CD4 sequences (listed in Table 2) as described above. The alignments were verified manually at amino acid level. Then the phylogenetic trees were reconstructed using neighbor-joining (NJ) method^52^ with MEGA6^30^ (Fig. 1a,b) and maximum-likelihood (ML) method with PhyML^53^ (Fig. S1A). Note that the phylogenetic trees of 58 primate tetherins reconstructed by these two methods yielded similar topology with partial difference in the relationships between certain tetherins; particularly mustached monkey, Red-eared monkey, and Sclater’s monkey. We assume that these minor differences are due to higher similarity among these species, because each species did not form a monophyletic cluster on the tree, and bootstrap support for most splits between the sequences were fairly low (< 75%, data not shown). Moreover, both NJ and ML trees were used for further PAML analyses, and the small topological difference between these two trees did not affect the results (Fig. 1c and S1B). Furthermore, we confirmed that the nucleotide sequence of the ancestral *Cercopithecini* tetherin inferred by NJ tree was identical to that by ML tree (data not shown). We then conducted the analysis to detect positive selection along the tree. To infer positive selection across various primate lineages, two pairs of site models implemented in the PAML package v 4.7^54^ were used to conduct the likelihood ratio tests for 58 tetherin genes (Fig. 1c,e), and 22 CD4 genes (Fig. 1d,f), respectively: M1 (neutral model) versus M2 (selection model) and M7 (neutral model) versus M8 (selection model). The REL method in HyPhy^55^ was also employed to detect positive selection (Fig. 3d). The ancestral *Cercopithecini* tetherin was inferred by using site model in the PAML analysis (Fig. 2a). Since we were particularly interested in whether the clades of tribe *Cercopithecini*, the hosts of *vpu*-negative SIVs, and those of *vpu*-positive SIVs have evolved under positive selection, we further focused on the 22 tetherins of this clade. First, the branch-site model in PAML was employed for the analysis. This model allows dN/dS ratio to vary both among sites and branches, which is very useful for detecting positive selection along a particular lineage or clade (pre-specified as foreground branches)^54^. In our analysis, all the 22 tetherins of tribe *Cercopithecini*, 14 tetherins of the hosts of *vpu*-negative SIVs, and 6 tetherins of the hosts of *vpu*-positive SIVs, were respectively specified as the foreground branches (Fig. 3a). Next, the site model in PAML and REL method in HyPhy were performed to these sequences (Fig. 3b–d). Moreover, the genetic distance (Fig. 3f) was calculated for the 14 tetherin genes of the hosts of *vpu*-negative SIV and the 6 genes of the hosts of *vpu*-positive SIV, respectively, by using MEGA6^30^. We computed the overall mean distance was computed by using Tamura-Nei model^56^ with 100 bootstrap replications.

### Protein homology modeling

The 3D structure of the ECD of ancestral *Cercopithecini* tetherin (Fig. 3e) was simulated by the Swiss-Model server (http://swissmodel.expasy.org/) using the crystal structure of the ECD of human tetherin (PDB code: 3MQB) as the template^31^.

### BEAST analysis

The full-genome sequences of 34 SIV strains (listed in Table 4) were retrieved from the HIV Sequences Database (http://www.hiv.lanl.gov/content/sequence). Then, the *gag, pol, env* and *vif* genes were extracted from each viral sequence and were respectively aligned using MAFFT^57^. The resulting alignments were manually verified at the amino acid level. We then performed the single breakpoint analysis^55^ implemented in the HyPhy package to test for the recombination in each aligned dataset. The results showed that no evidence of recombination was detected in all four analyses. We further performed Gblocks (http://molevol.cmima.csic.es/castresana/Gblocks_server.html) v 9.1b to remove poorly aligned regions from these alignments. Finally, a concatenated dataset of all four genes was created by using BioEdit (http://www.mbio.ncsu.edu/bioedit/bioedit.html) v 7.2.5. To infer the time of most recent common ancestors of these SIVs, a Bayesian approach implemented in the BEAST package (http://beast.bio.ed.ac.uk) v 1.7.5^58^ was employed. This analysis was conducted at the amino acid level, as the 34 SIV strains were highly divergent. We used a JTT substitution model with gamma-distributed rate variation among sites. The uncorrelated lognormal relaxed molecular clock model was employed to estimate substitution rates and the Yule process of speciation was used for the tree prior. We specified a uniform distributed prior (30,000–130,000 yr, initial = 70,000 yr) for the age of the root of the tree^59^. Five independent Markov Chain Monte Carlo (MCMC) analyses were run for 10–20 million generations with sampling every 1000 generations. We then used the program Tracer (tree.bio.ed.ac.uk/software/tracer) v 1.6 to check for the convergence and to confirm that the effective sample size (ESS) value was higher than 200 for all runs. The maximum clade credibility (MCC) tree was generated by summarizing the sample of trees produced by BEAST after a 10% burn-in using the TreeAnnotator program v 1.7.5, and the resulting MCC tree was viewed using FigTree (http://tree.bio.ed.ac.uk/) v 1.4.2.

### Plasmid construction

The HA-tagged Nef expression plasmids of SIVgsn (strain 99CM166), SIVmon (strains 99CMCML1 and NG1), (SIVmus strains 01CM1085 and 01CM1239) were used in our previous study^25^. The Nef open reading frames (ORFs) of SIVden (strain CD1), SIVagmSab (strain SAB1), SIVsyk (strain KE44), SIVlhoest (strain LHO7), SIVsun (strain L14), SIVtal (strain 00CM266), and SIVdeb (strains CM5 and Kin1) were obtained from GeneArt Gene Synthesis service (Life Technologies). The Nef ORF of SIVagmVer strain TYO1 was obtained by PCR using pSA212 (an infectious molecular clone of SIVagmVer strain TYO1)^60^ as the template and the following primers: TYO1 Nef-Fwd, 5′-TTT TTT TTT CTA GAA TGG GCT CGC AGA ACT CA-3′; TYO1 Nef-Rev, 5′-TAT ATA TAT ATA GAT ATC CTT CCT CTT CAC CAG CC-3′. The resultant DNA fragment was digested with XbaI and EcoRV and was inserted into the XbaI-EcoRV site of pCGCG vector.

### Cell culture and transfection

HEK293T cells and TZM-bl cells (obtained through NIH AIDS Research and Reference Reagent Program) were maintained in Dulbecco’s modified Eagle medium (Sigma) containing 10% heat-inactivated FCS and antibiotics. Transfection was performed by using PEI Max (GE Healthcare) according to the manufacturer’s protocol. Various amounts of KGC-tagged ancestral *Cercopithecini* tetherin expression plasmids (0, 10, 40 ng) and pNL4-3Δ*vpu*Δ*nef* (1,200 ng) were cointroduced with or without respective SIV Nef expression plasmid (400 ng) into HEK293T cells. At 48 hours post-transfection, the culture supernatants and transfected cells were harvested and were respectively used for TZM-bl assay and Western blotting as described below.

### Western blotting and TZM-bl assay

The culture supernatant harvested at 48 hours post-transfection was centrifuged to remove cells and produce virus suspensions. The infectivity of virus suspensions was measured by TZM-bl assay as previously described^25^. Briefly, 100 μl of the virus solution was inoculated into TZM-bl cells in 96-well plate (Nunc), and the β-galactosidase activity was measured by using the Galacto-Star mammalian reporter gene assay system (Applied Biosystems) and a 2030 ARVO X multilabel counter instrument (PerkinElmer) according to the manufacturers’ procedure. Western blotting was performed as previously described^25^ by using the following antibodies: anti-p24 polyclonal antibody (ViroStat), anti-KGC antibody (clone 21B10; Medical and Biological Laboratories, Inc.), anti-HA antibody (3F10; Roche), and anti-alpha-Tubulin (TUBA) antibody (DM1A; Sigma).

### Statistical analyses

The data expressed as average with standard error (Fig. 3f) or standard deviation (Fig. 4b), and significant differences were determined by Student’s *t* test (Fig. 4b) or Welch’s *t* test (Fig. 5c).

## Additional Information

**How to cite this article**: Takeuchi, J. S. *et al.* Coevolutionary dynamics between tribe *Cercopithecini* tetherins and their lentiviruses. *Sci. Rep.* **5**, 16021; doi: 10.1038/srep16021 (2015).

## Supplementary Material

Supplementary Information

## Figures and Tables

**Table 1:** Accession numbers of primate tetherin used in this study.

Family/infraorder^1^	Common name^2^	Scientific name	Accession number^3^
Hominidae (Hominoids)	Human	Homo sapiens	AK223124
	Human	Homo sapiens	NM_004335
	Chimpanzee	Pan troglodytes	NM_001190480
	Bonobo	Pan paniscus	HM136907
	Bonobo	Pan paniscus	XM_003817802
	Gorilla	Gorilla gorilla	GQ925926
	Gorilla	Gorilla gorilla	HM136906
	Gorilla	Gorilla gorilla	XM_004060266
	Orangutan	Pongo pygmaeus	HM136908
	Orangutan	Pongo abelii	NM_001172587
	Gibbon	Hylobates agilis	HM136910
	Gibbon	Nomascus leucogenys	HM136909
Cercopithecidae (OWMs)	Mustached monkey	Cercopithecus cephus	GQ864267
	Dent’s mona monkey	Cercopithecus denti	HE680870
	Red-eared monkey	Cercopithecus erythrotis	LC012317^4^
	Sclater’s monkey	Cercopithecus sclateri	LC012319^4^
	Sclater’s monkey	Cercopithecus sclateri	LC012320^4^
	Red-eared monkey	Cercopithecus erythrotis	LC012316^4^
	Mustached monkey	Cercopithecus cephus	LC012318^4^
	Red-tailed monkey	Cercopithecus ascanis	LC012315^4^
	Mustached monkey	Cercopithecus cephus	GQ925925
	Sykes’ monkey	Cercopithecus albogularis	LC012321^4^
	Sykes’ monkey	Cercopithecus albogularis	LC012322^4^
	Sun-tailed monkey	Cercopithecus solatus	LC012323^4^
	L’Hoest’s monkey	Cercopithecus lhoesti	LC012313^4^
	Mona monkey	Cercopithecus mona	GQ925924
	Campbell’s mona monkey	Cercopithecus campbelli	LC012314^4^
	Greater spot-nosed monkey	Cercopithecus nictitans	GQ925923
	De Brazza’s monkey	Cercopithecus neglectus	HE680871
	Talapoin monkey	Miopithecus talapoin	HM136913
	Patas monkey	Erythrocebus patas	HM136911
	Tantalus monkey	Chlorocebus tantalus	FJ345303
	Grivet monkey	Chlorocebus aethiops	FJ943430
	Grivet monkey	Chlorocebus aethiops	HM136912
	Rhesus macaque	Macaca mulatta	FJ943431
	Rhesus macaque	Macaca mulatta	FJ943432
	Rhesus macaque	Macaca mulatta	GQ304749
	Rhesus macaque	Macaca mulatta	HM136914
	Rhesus macaque	Macaca mulatta	HM775182
	Rhesus macaque	Macaca mulatta	NM_001161666
	Pig-tailed macaque	Macaca nemestrina	FJ914988
	Pig-tailed macaque	Macaca nemestrina	FJ914989
	Red-capped mangabey	Cercocebus torquatus	AB907706
	Red-capped mangabey	Cercocebus torquatus	AB907707
	Sooty mangabey	Cercocebus atys	FJ864713
	Sooty mangabey	Cercocebus atys	FJ864714
	Olive Baboon	Papio anubis	XM_003915138
	Red-shanked douc langur	Pygathrix nemaeus	HM136916
	Francois’ leaf monkey	Trachypithecus francoisi	HM136917
	Colobus monkey	Colobus guereza	HM136915
Platyrrhini (NWMs)	Night monkey	Aotus lemurinus	FJ638414
	Night monkey	Aotus vociferans	FJ638417
	Night monkey	Aotus vociferans	FJ638418
	Night monkey	Aotus vociferans	FJ638415
	White-faced saki monkey	Pithecia pithecia	HM136920
	White-lipped tamarin	Saguinus labiatus	HM136918
	Common woolly monkey	Lagothrix lagotricha	HM136922
	Bolivian red howler monkey	Alouatta sara	HM136921

^a^Family (Hominidae and Cercopithecidae) and infraorder (Platyrrhini) are presented in italic. Popular name of each family/infraorder is presented in parenthesis. OWMs, old world monkeys; NWMs, new world monkeys.

^b^The common name of each primate is identical to that in Fig. 1a.

^c^The GenBank accession numbers (http://www.ncbi.nlm.nih.gov/genbank/) of tetherins are listed.

^d^The newly identified sequences in this study.

**Table 2:** Accession numbers of primate CD4 used in this study.

Family/infraorder^a^	Common name^b^	Scientific name	Accession number^c^
Hominidae (Hominoids)	Human	Homo sapiens	AK312828
	Human	Homo sapiens	BC025782
	Human	Homo sapiens	BT019791
	Human	Homo sapiens	BT019811
	Human	Homo sapiens	NM_000616
	Chimpanzee	Pan troglodytes	EF437437
	Chimpanzee	Pan troglodytes	EF437438
	Chimpanzee	Pan troglodytes	EF437439
	Chimpanzee	Pan troglodytes	EF437441
	Chimpanzee	Pan troglodytes	EF437442
	Chimpanzee	Pan troglodytes	NM_001009043
	Gorilla	Gorilla gorilla	XM_004052582
	Gibbon	Nomascus leucogenys	XM_004092147
	Gibbon	Nomascus leucogenys	XM_004092148
Cercopithecidae (OWMs)	Mustached monkey	Cercopithecus cephus	LC017837^d^
	Grivet monkey	Chlorocebus aethiops	D86589
	Rhesus macaque	Macaca mulatta	D63347
	Pig-tailed macaque	Macaca nemestrina	D63346
	Sooty mangabey	Cercocebus atys	KP406148^d^
	Sooty mangabey	Cercocebus atys	KP406149^d^
Platyrrhini (NWMs)	Night monkey	Aotus nancymaae	FJ623078
	Marmoset	Callithrix jacchus	NM_001267772

^a^Family (Hominidae and Cercopithecidae) and infraorder (Platyrrhini) are presented in italic. Popular name of each family/infraorder is presented in parenthesis.

^b^The common name of each primate is identical to that in Fig. 1b.

^c^The GenBank accession numbers (http://www.ncbi.nlm.nih.gov/genbank/) of CD4s are listed.

^d^The newly identified sequences in this study.

**Table 3:** Divergence times and node support for SIVs in Fig. 5a.

Node^a^	Date (Years ago)	Date 95% HPD		Posterior Probability	ML bootstrap value^b^
1	2941	1499	6932	1.00	100
2	3525	1722	8098	1.00	100
3	7319	4247	16908	1.00	98
4	2656	1296	6073	1.00	100
5	9784	5809	22301	1.00	67
6	12242	7015	27055	1.00	100
7	2487	1166	5889	1.00	100
8	19418	11648	44898	1.00	84
9	7662	3852	17731	1.00	100
10	22652	13735	52774	1.00	83
11	3407	1750	8020	1.00	100
12	8129	4322	18855	1.00	100
13	19218	10862	44407	1.00	100
14	25397	15346	58954	1.00	100
15	2634	1305	5968	1.00	97
16	4184	4184	9774	1.00	100
17	10544	5952	23873	1.00	77
18	13030	7494	29962	1.00	100
19	18345	10406	41995	1.00	100
20	30483	18224	69491	1.00	100
21	1111	553	2587	1.00	100
22	3256	1744	7517	1.00	97
23	4521	2535	10510	1.00	100
24	3547	1715	8323	1.00	100
25	10063	5773	23593	1.00	100
26	21134	12230	49699	1.00	100
27	2990	1354	7004	1.00	100
28	7239	3519	16469	1.00	100
29	28102	16712	65288	1.00	100
30	35766	22954	81980	0.74	100
31	5749	2825	12861	1.00	83
32	9047	5188	20893	1.00	100
Root	40894	30004	92529	NA^c^	NA^c^

^a^Each node is correspond to that in Fig. 5a.

^b^Bootstrap value is obtained from ML tree.

^c^NA, not applicable.

**Table 4:** Accession numbers of SIV used in this study.

Virus^a^	Strain^a^	Host^b^		Accession number^c^
SIVmus	01CM1085	Mustached monkey	(*Cercopithecus cephus*)	AY340700
SIVmus	01CM1239	Mustached monkey	(*Cercopithecus cephus*)	EF070330
SIVmus	01CM2500	Mustached monkey	(*Cercopithecus cephus*)	EF070331
SIVmus	01CM1246	Mustached monkey	(*Cercopithecus cephus*)	EF070329
SIVgsn	99CM166	Greater spot-nosed monkey	(*Cercopithecus nictitans*)	AF468659
SIVgsn	99CM71	Greater spot-nosed monkey	(*Cercopithecus nictitans*)	AF468658
SIVmon	99CMCML1	Mona monkey	(*Cercopithecus mona*)	AY340701
SIVtal	00CM266	Talapoin monkey	(*Miopithecus talapoin*)	AY655744
SIVtal	01CM8023	Talapoin monkey	(*Miopithecus talapoin*)	AM182197
SIVsyk	173	Sykes’ monkey	(*Cercopithecus albogularis*)	L06042
SIVsyk	KE51	Sykes’ monkey	(*Cercopithecus albogularis*)	AY523867
SIVdeb	04CMPF3061	De Brazza’s monkey	(*Cercopithecus neglectus*)	FJ919724
SIVdeb	CM40	De Brazza’s monkey	(*Cercopithecus neglectus*)	AY523865
SIVdeb	CM5	De Brazza’s monkey	(*Cercopithecus neglectus*)	AY523866
SIVden	CD1	Dent’s mona monkey	(*Cercopithecus denti*)	AJ580407
SIVagmVer	155	Vervet monkey	(*Chlorocebus pygerythrus*)	M29975
SIVagmVer	AGM3	Vervet monkey	(*Chlorocebus pygerythrus*)	M30931
SIVagmVer	TYO1	Vervet monkey	(*Chlorocebus pygerythrus*)	AB253736
SIVagmGri	677	Grivet monkey	(*Chlorocebus aethiops*)	M58410
SIVagmTan	TAN1	Tantalus monkey	(*Chlorocebus tantalus*)	U58991
SIVagmSab	SAB1	Green monkey	(*Chlorocebus sabaeus*)	U04005
SIVlhoest	447	L’Hoest’s monkey	(*Cercopithecus lhoesti*)	AF188114
SIVlhoest	485	L’Hoest’s monkey	(*Cercopithecus lhoesti*)	AF188115
SIVlhoest	524	L’Hoest’s monkey	(*Cercopithecus lhoesti*)	AF188116
SIVlhoest	LHO7	L’Hoest’s monkey	(*Cercopithecus lhoesti*)	AF075269
SIVsun	K08_Gab	Sun-tailed monkey	(*Cercopithecus solatus*)	FR751162
SIVsun	L14	Sun-tailed monkey	(*Cercopithecus solatus*)	AF131870
SIVmnd1	GB1	Mandrill	(*Mandrillus sphinx*)	M27470
SIVwrc	97CI14	Western red colobus	(*Piliocolobus badius*)	AM745105
SIVwrc	98CI04	Western red colobus	(*Piliocolobus badius*)	AM713177
SIVwrc	Pbt-05GM-X02	Western red colobus	(*Piliocolobus badius*)	AM937062
SIVcol	BMC01	Black-and-white colobus	(*Colobus guereza*)	KF214240
SIVcol	CGU1	Black-and-white colobus	(*Colobus guereza*)	AF301156
SIVcol	BMC07	Black-and-white colobus	(*Colobus guereza*)	KF214241

^a^The names of virus and strain are identical to that in Fig. 5a.

^b^The common name and scientific name (in parenthesis) of the host monkey of each SIV are presented.

^c^The GenBank accession numbers (http://www.ncbi.nlm.nih.gov/genbank/) of SIVs are listed.
