# Supplementary material for: Coevolutionary dynamics between tribe Cercopithecini tetherins and their lentiviruses
Source: Sci Rep. 2015 Nov 4;5:16021. doi: 10.1038/srep16021 (PMC4631996; doi:10.1038/srep16021)
Supplement: Supplementary Information [file srep16021-s1.pdf]

Coevolutionary dynamics between tribe *Cercopithecini* tetherins and their lentiviruses

Junko S. Takeuchi, Fengrong Ren, Rokusuke Yoshikawa, Eri Yamada, Yusuke Nakano, Tomoko Kobayashi, Kenta Matsuda, Taisuke Izumi, Naoko Misawa, Yuta Shintaku, Katherine S. Wetzel, Ronald G. Collman, Hiroshi Tanaka, Vanessa M. Hirsch, Yoshio Koyanagi, and Kei Sato

Supplementary Information:

Supplementary Figure 1: ML phylogenetic tree and related analysis
Supplementary Figure 2: correlation between viral infectivity and antigen in supernatant
Supplementary Figure 3: original (uncropped) blots

a

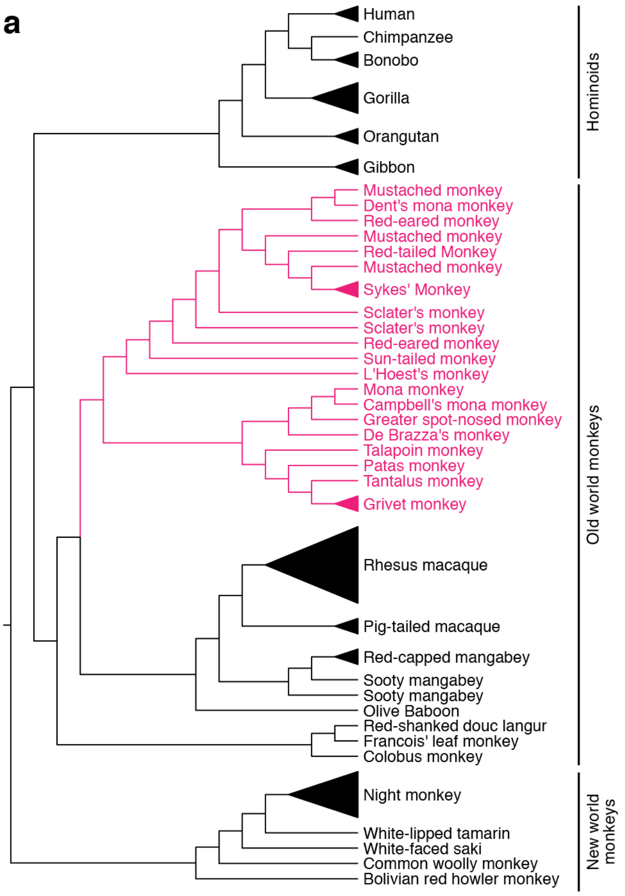

b

Primate tetherin (n=58)

Region analyzed (amino acid)	$2\Delta\ell$	*P* value
**Full gene (1-191)**	**43.41**	**< 0.001**
**Cytoplasmic tail (1-25)**	**26.76**	**< 0.001**
**Transmembrane domain (26-55)**	**10.81**	**< 0.005**
Extracellular domain (56-191)	ND	

Supplementary Figure 1. Maximum likelihood (ML) tree and related analyses. – Related to Figure 1.

(a) A phylogenic tree of 58 primate tetherins reconstructed using ML method. The tree was rerooted with the New World monkey clade. The species belonging to Tribe *Cercopithecini* are shown in pink. GenBank accession numbers are listed in Table 1. (b) The positive selection detected in different regions of tetherin. The regions inferred to be under positive selection with statistical significance are represented in bold. ND, not detected.

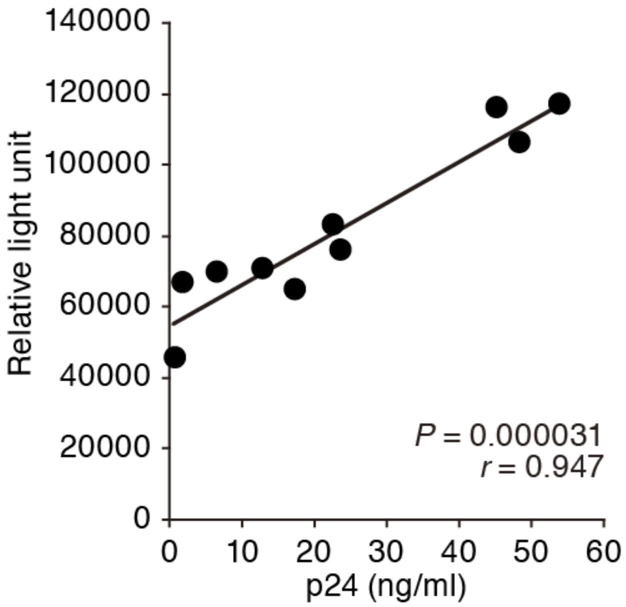

Supplementary Figure 2. Correlation between viral infectivity and antigen in culture supernatant. – Related to Figure 4.

Correlation between the levels of viral infectivity and antigen detected in the culture supernatants of the transfected cells. Viral infectivity (y-axis) was measured by TZM-bl assay, and the data is shown as relative light unit. The amount of viral p24 antigen (x-axis) was measured by ELISA. Each dot represents the value of respective virus solution. Pearson correlation coefficient (*r*) and the *P* value are indicated.

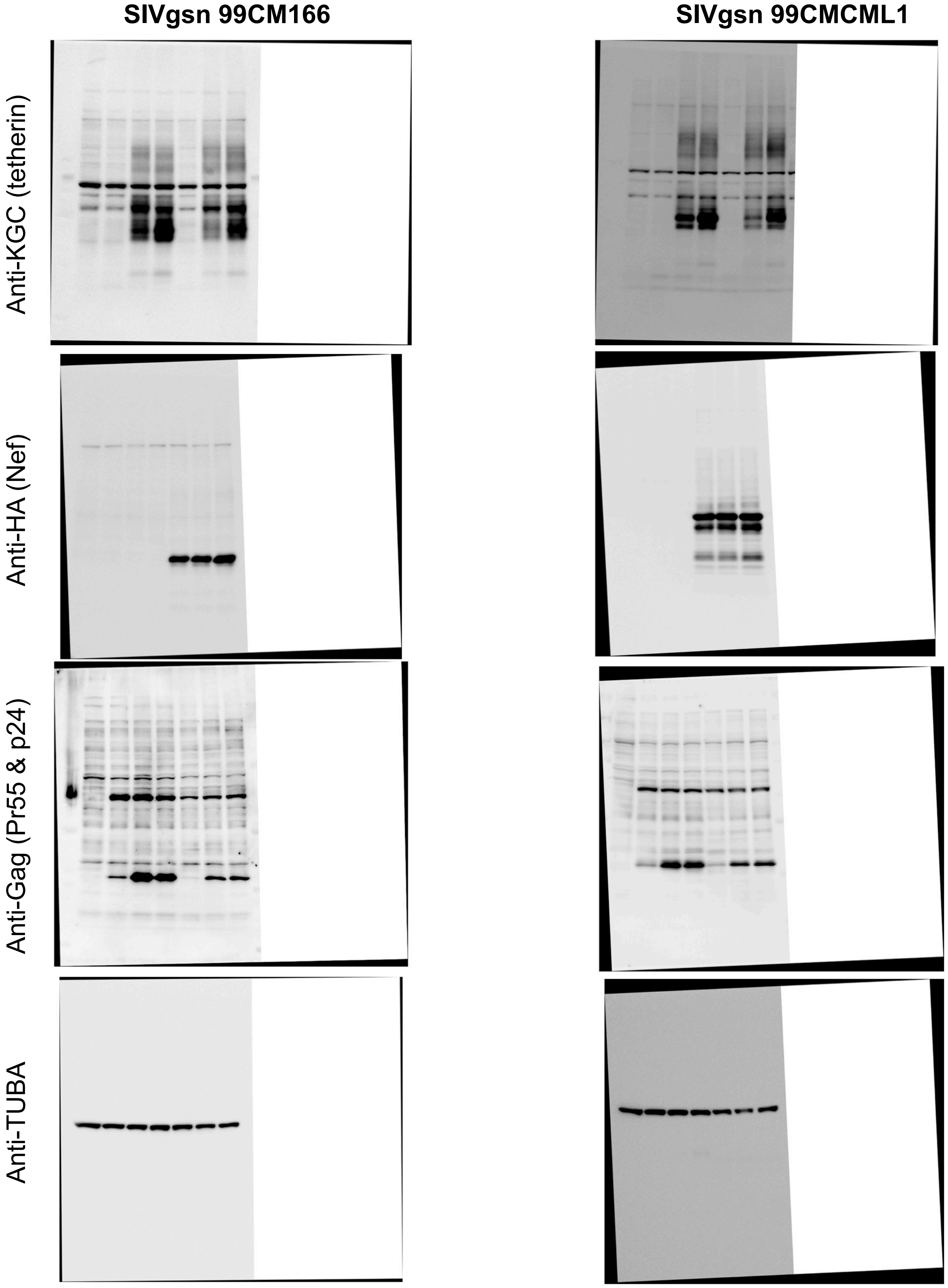

Supplementary Figure 3. Original (uncropped) blots of Figure 4a.

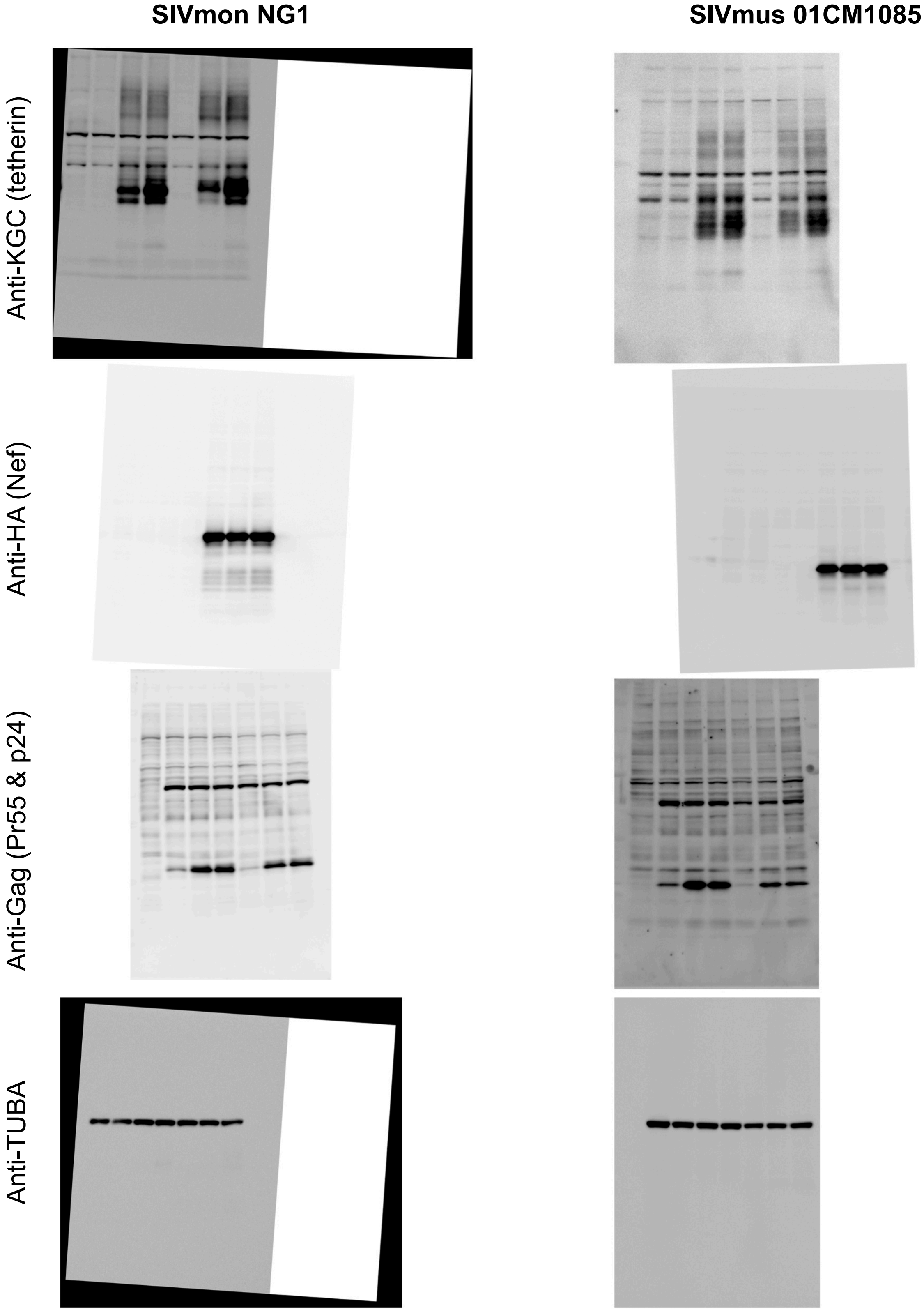

Supplementary Figure 3. Original (uncropped) blots of Figure 4a. (continued)

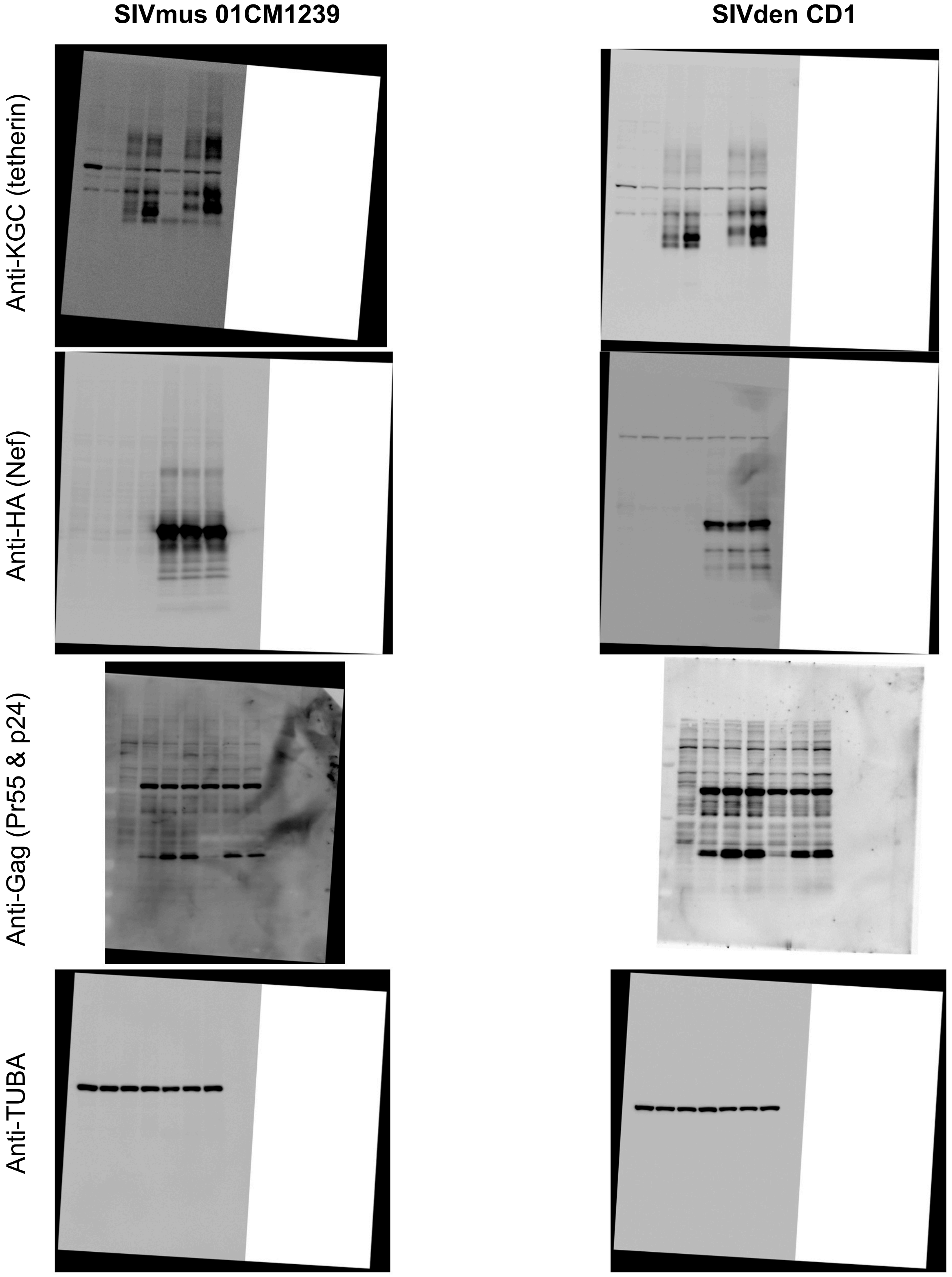

Supplementary Figure 3. Original (uncropped) blots of Figure 4a. (continued)

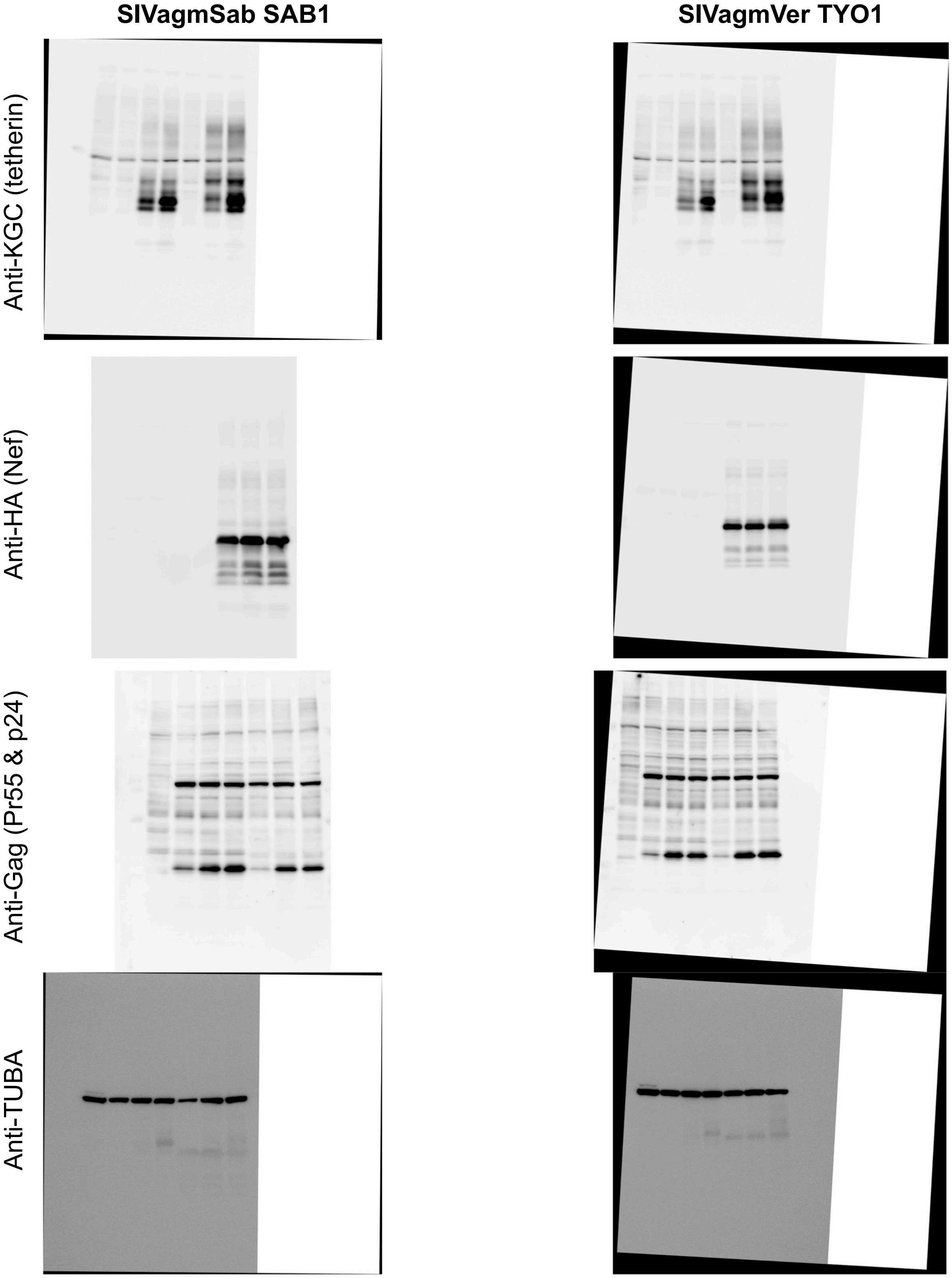

Supplementary Figure 3. Original (uncropped) blots of Figure 4a. (continued)

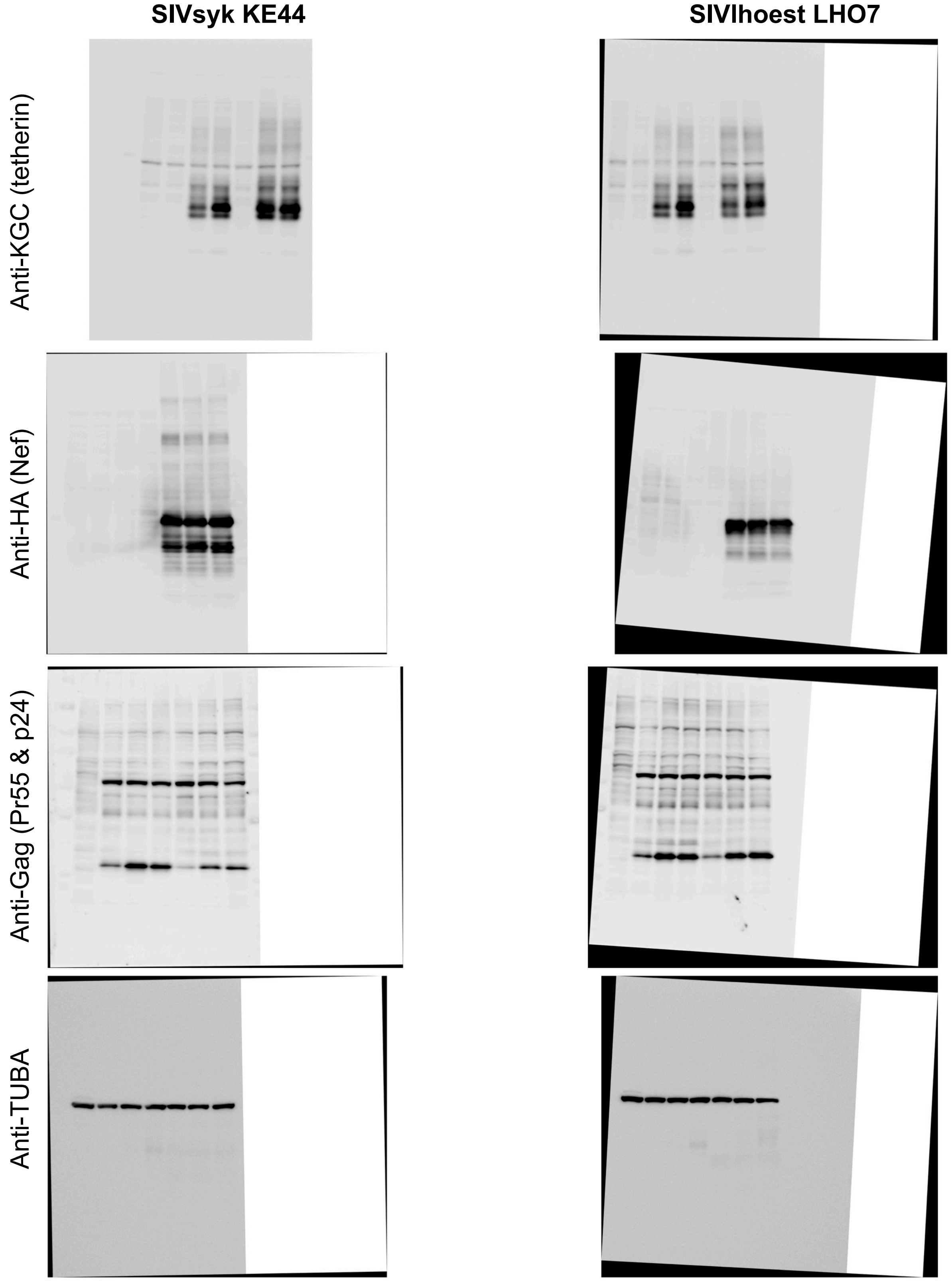

Supplementary Figure 3. Original (uncropped) blots of Figure 4a. (continued)

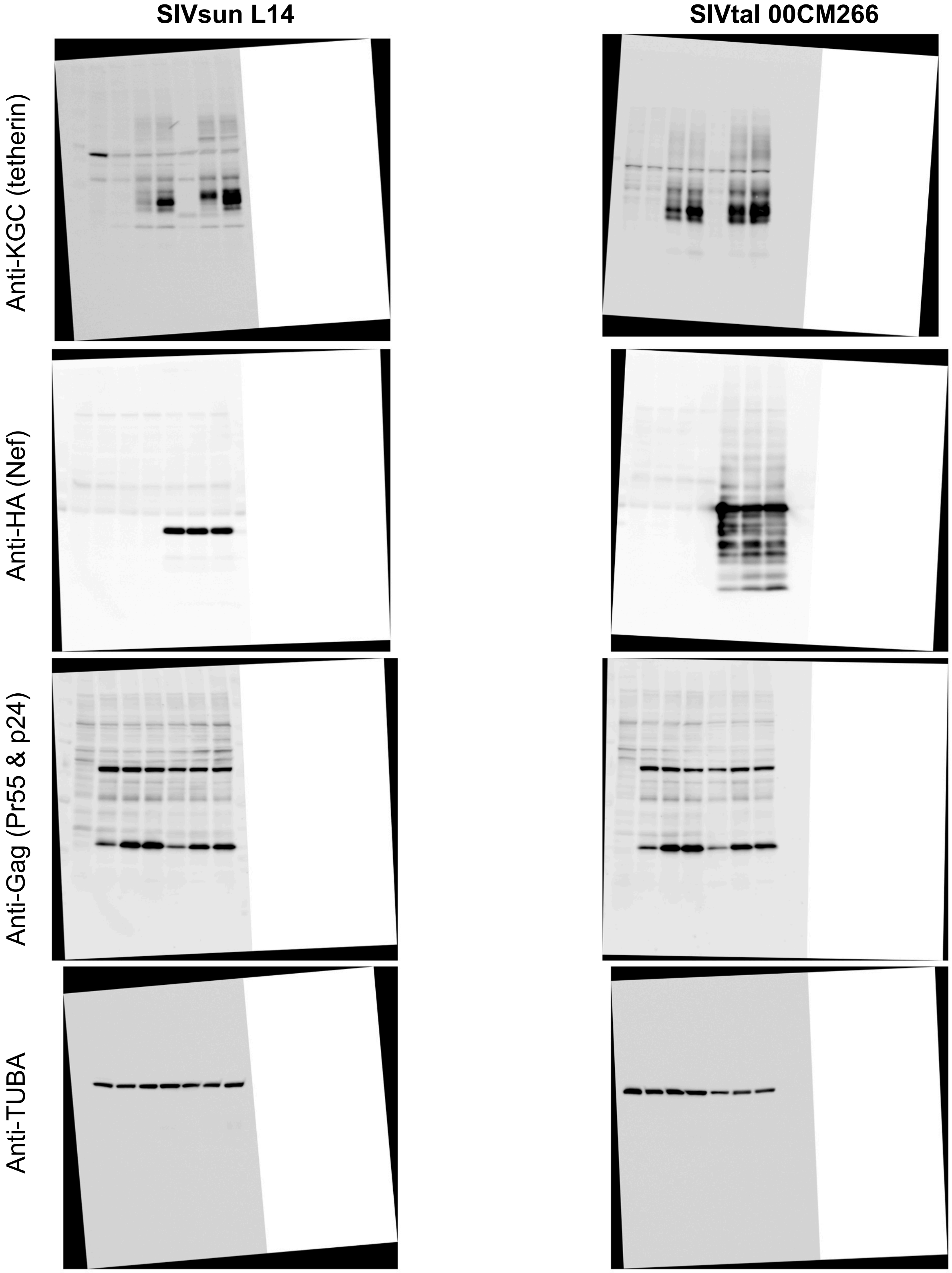

Supplementary Figure 3. Original (uncropped) blots of Figure 4a. (continued)

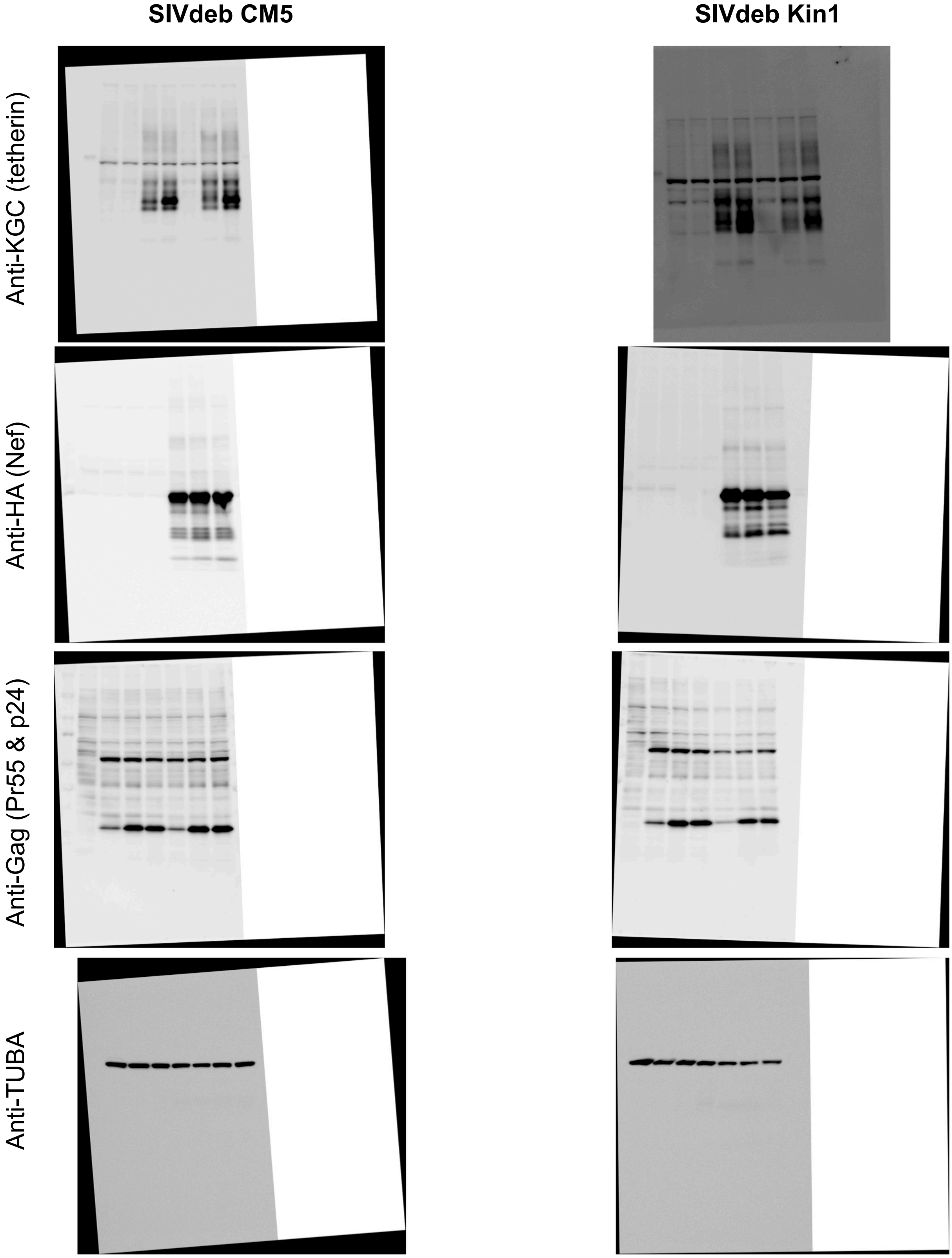

Supplementary Figure 3. Original (uncropped) blots of Figure 4a. (continued)
